# A Study on the Design of Knee Exoskeleton Rehabilitation Based on the RFPBS Model

**DOI:** 10.3390/biomimetics9070410

**Published:** 2024-07-05

**Authors:** Qiujian Xu, Junrui Li, Nan Jiang, Xinran Yuan, Siqi Liu, Dan Yang, Xiubo Ren, Xiaoyu Wang, Mingyi Yang, Yintong Liu, Peng Zhang

**Affiliations:** 1 School of Arts and Design, Yanshan University, Haigang District, Qinhuangdao 066000, China; xuqiujian@ysu.edu.cn (Q.X.); lijunrui_1023@163.com (J.L.); 2021327070@knu.ac.kr (N.J.); yuanxinran0112@163.com (X.Y.); liusiqi19990919@163.com (S.L.); 2 Department of Design, Kyungpook National University, Daegu 41566, Republic of Korea; 3 School of Public Performing Arts, Catholic University of Daegu, Gyeongsan 38430, Republic of Korea; yangdan6413@163.com (D.Y.); xiuboren@gmail.com (X.R.); wangxiaoyu_9607@163.com (X.W.); yangmingyi717@163.com (M.Y.); 13831481889@163.com (Y.L.)

**Keywords:** knee exoskeleton, RFPBS model, process knowledge representation, simulation analysis

## Abstract

The gait rehabilitation knee exoskeleton is an advanced rehabilitative assistive device designed to help patients with knee joint dysfunction regain normal gait through training and activity support. This paper introduces a design framework based on the process knowledge representation method to optimize the design and control efficiency of the knee exoskeleton. This framework integrates knowledge of design objects and processes, specifically including requirements, functions, principle work areas, and the representation and multi-dimensional dynamic mapping of the Behavior–Structure (RFPBS) matrix, achieving multi-dimensional dynamic mapping of the knee exoskeleton. This method incorporates biomechanical and physiological knowledge from the rehabilitation process to more effectively simulate and support gait movements during rehabilitation. Research results indicate that the knee rehabilitation exoskeleton design, based on the RFPBS process knowledge representation model, accomplishes multi-dimensional dynamic mapping, providing a scientific basis and effective support for the rehabilitation of patients with knee joint dysfunction.

## 1. Introduction

With the rapid development of society and the economy, the proportion of China’s aging population suffering from lower limb activity disorders has been rising, especially knee dysfunction patients [1,2,3,4,5]. To meet this challenge, the development of knee exoskeletons has emerged as a key technology to address gait rehabilitation issues [6,7,8]. The technology integrates the latest achievements in the fields of human biomechanics [9,10,11,12], mechanical design [10,13,14,15,16], bionics [17,18], electromechanical control [10,16], and information processing [18,19,20,21,22], and together with intelligent robotics [23,24], it not only provides patients with walking assistance, but also targeted training gait, aiming at restoring and enhancing patients’ knee joint function and musculoskeletal strength [25,26,27,28]. In this context, it is particularly important to develop a design method based on process knowledge representation to guide the design of knee exoskeletons. However, current research is still insufficient in integrating design knowledge and problems solving strategies, so the Requirement–Function–Principle–Behavior–Structure (RFPBS) process knowledge representation model was constructed. The model utilizes the principle of biomechanics and, through the optimized matching between function and structure, not only helps to improve the quality of the research and development of the knee exoskeleton but also enhances the coordination and comfort of the human–computer coupled system, which provides a solid theoretical and empirical foundation for future design applications.

In this study, the Requirement–Function–Principle–Behavior–Structure (RFPBS) process knowledge representation model is proposed, aimed at effectively addressing the complex challenges in product design. Compared to the traditional Function–Behavior–Structure (FBS) model, the RFPBS model enhances the knowledge modeling process by incorporating user requirements and principle space constraints, thereby facilitating a more thorough and rational design knowledge modeling process and achieving innovative multi-level mapping solutions. The RFPBS model systematically captures and organizes the tacit knowledge of designers throughout the design process, ensuring its comprehensibility to both human designers and computer systems. Moreover, based on the theory of design knowledge flow, the model explores methods for product module decomposition driven by user requirements and establishes a support system for configuring knee orthosis modules. By clearly defining the key elements of the design process—requirements, functions, principles, behaviors, and structures—the RFPBS model provides a clear framework for design activities and builds a scientifically sound product design support platform. Through multi-angle and hierarchical module segmentation and identification methods, this model achieves precise mapping relationships from user requirements to product structures, promoting multi-level innovative designs focused on function decoupling and structure–function matching. This structured approach not only effectively identifies and records valuable design knowledge but also fosters the logical organization and effective transmission of design knowledge. In particular, the model enhances the systematic integration of design knowledge by closely aligning the detailed features of design objects with the process of design activities. The introduction of the RFPBS model significantly improves the quality and efficiency of design decisions by clearly specifying the inputs and outputs at each stage of the design process, helping designers systematically understand and apply engineering knowledge, thus advancing innovation and optimization in product design. Furthermore, the implementation of the RFPBS model provides a solid theoretical and practical framework for the development of design knowledge management software, enabling the creation of systems that not only record design data but also capture and reuse key knowledge generated during the design process. This capability greatly enhances the collaborative efficiency and innovative capacity of design teams, providing strong support for the multidimensional integration of complex system designs. Therefore, the proposal and implementation of the RFPBS model hold significant theoretical and practical value for enhancing the scientific and systematic nature of engineering design and achieving efficient and innovative design solutions.

## 2. Theoretical Background

In product design, the design process itself is an organizational activity aimed at integrating the resources of all stakeholders to achieve clear objectives. Throughout this process, knowledge is continually acquired and updated among stakeholders and plays a role in the ongoing changes in design activities. Particularly in the study of the key technical layers of product design, understanding and analyzing the flow of design knowledge is crucial. This includes understanding how to effectively transfer knowledge between people and knowledge processing organizations, and how to build and strengthen the network framework of design knowledge through effective knowledge flow. In the key technical layers of product design, the fluidity of knowledge resources is especially important. It flows not only between the user layer and the technical layer but also greatly enhances the product innovative design process through the driving force of knowledge and collaborative sharing. Effectively managing and utilizing these knowledge resources can significantly enhance the efficiency of knowledge utilization in product design through in-depth analysis and research by designers, thereby achieving the goal of innovative design. This methodology emphasizes the central role of knowledge resources in the key technical layers of product design, as well as the crucial position of knowledge management in promoting the improvement of design quality and efficiency.

### 2.1. Product Design Key Technology Layer Study

#### 2.1.1. Establishment of the Design Knowledge Network Framework

**Figure 1:**
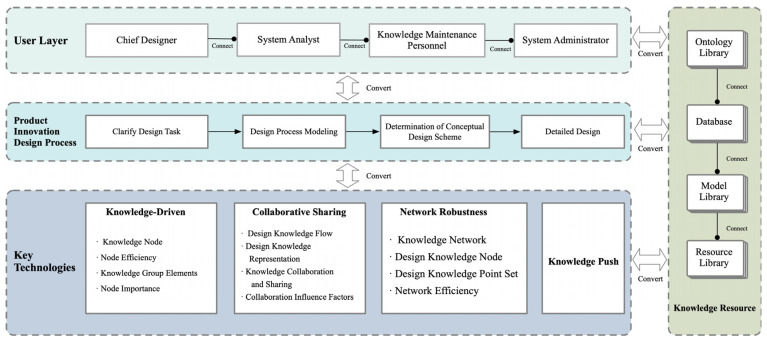
Knowledge Network Framework for Product Innovation Design.

In the field of product design, the design process begins with an ambiguous conceptual phase and progresses towards a predetermined goal with its inherent complexity and iterative nature [19,29,30]. The process moves from original sketches to detailed design, reflecting the evolution of a system from initial uncertainty to final clarity [31,32]. The core task is to gradually transform the designer’s initial concepts into an organized logical framework through which design goals can be achieved, and systematically structuring design knowledge and understanding how it is expressed is essential for optimizing the design process. In addition, design activity is also an organizational process that integrates the resources of various stakeholders, in which the flow and update of knowledge resources is continuous. Knowledge resources, as the basis of innovative design, flow between the user and technical levels, and the driving and sharing of knowledge energizes product innovation, improves the efficiency of design knowledge utilization, and thus facilitates the innovation and development of product design, as shown in Figure 1.

**Figure 2:**
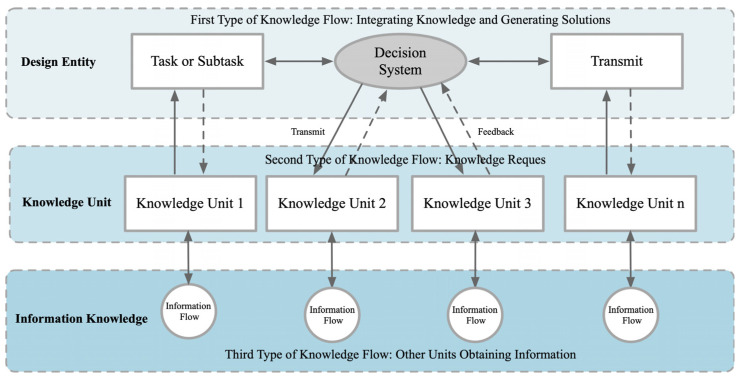
Hierarchical model of the design process.

The construction of a conceptual design model is ultimately a description of a functional problem that requires a set of feasible models to solve it, and the purpose of a process knowledge representation model is to identify the commonalities behind a series of elements, summarize them, generalize them, and condense them into a simple structure that can be reused and migrated [33]. This structure is then adapted and applied to different scenarios to improve the efficiency of design solution construction. In the product design process, the hierarchical model of mapping relationships is constructed through the interaction between design entities and knowledge units, which is specifically embodied in the bottom-up and top-down mapping mechanisms. That is, multiple modular systems integrate information into a single design entity and a single modular system decentralizes its information to multiple design entities. This bi-directional mapping not only facilitates the effective transfer between design entities, knowledge units, and information knowledge, but also enhances the systematic integration and application of information in the design process, thus forming a multi-level, highly interactive design knowledge network. This model not only clarifies the path of information flow, but also provides theoretical support and practical application for design decisions, as shown in Figure 2.

#### 2.1.2. Research Related to Knowledge Representation Modeling

For more than three decades, researchers have been trying to find better ways to formulate design as a comprehensive iterative process, and scholars have been working on FBS [34] for describing the process of rationalizing product design and development in the field of engineering, as well as coordinating the different design variables in order to achieve innovative product designs(See Table 1). Building on these basic models, further research has added more dimensions to address more specific issues. The Situated FBS [35] framework was introduced to extend the FBS model to help better understand design in an open, dynamic world. The RFBS [36] model incorporates requirements analysis as an important element of the FBS model and proposes its integration into design methodologies and modeling languages. In addition, the FCBS [37] model seeks a complementary combination of functional and case-based modeling to develop conceptual design support tools. A review of past research and its development reveals that the main focus has always been on the functional, behavioral, and structural elements of engineering design. In contrast to previous models, the RFPBS model proposed in this study innovatively incorporates the consideration of user requirements and workspace principles while focusing on the knowledge capture and representation of the complete design process.

## 3. Method

To overcome the limitations of existing conceptual design process models, this study proposes a new conceptual module design process model, the RFPBS model, to scientifically guide product design practices. This method, by introducing “ontology” as a design paradigm, optimizes the transformation of requirements and the construction of functional organizational structures, thereby facilitating the precise expression and management of design knowledge. This research emphasizes the importance of constructing effective methods to improve design efficiency and lay the foundation for future innovations, demonstrating the central role of scientific methodology in advancing design practice and theoretical development.

### 3.1. Model Construction

#### 3.1.1. Process Knowledge Solving Model

**Figure 3:**
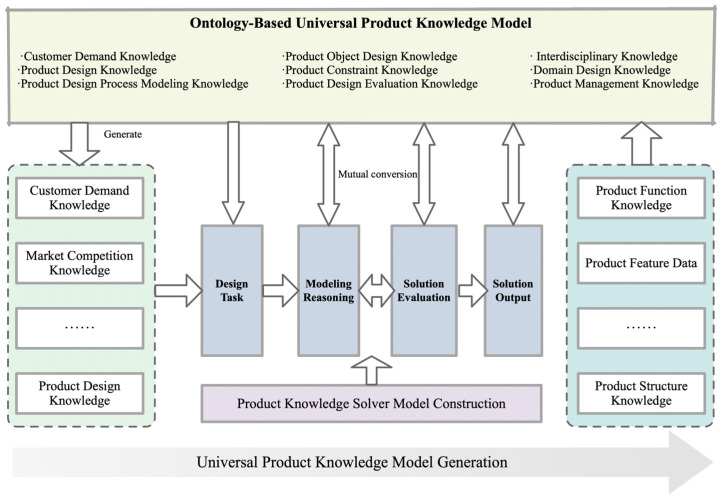
Ontology-based knowledge solving model.

Product design is a complex and iterative activity that often lacks clear definitions [38]. During the design process, designers need to refine the requirements and specifications of a product through multiple iterations. The process involves the transformation of the internal state of the system, which leads to the reconfiguration and solving of the structure and optimizes the efficiency of the transformation from input to output. System optimization requires a reconsideration of the relationships between function and form, and how these relationships drive the evolution of a product from concept to realization. Within this framework, an ontology-based design knowledge solving model becomes a key mechanism for driving product innovation. The model takes user requirements as a starting point, integrates product constraints, features, and structural knowledge, and stores them in a knowledge base. This approach allows designers to generate and evaluate multiple product structural solutions in order to select the optimal innovative design solution. The process not only systematically applies design-related knowledge, but also significantly improves the efficiency and innovativeness of design decisions through accurate model reasoning, as shown in Figure 3.

**Figure 4:**
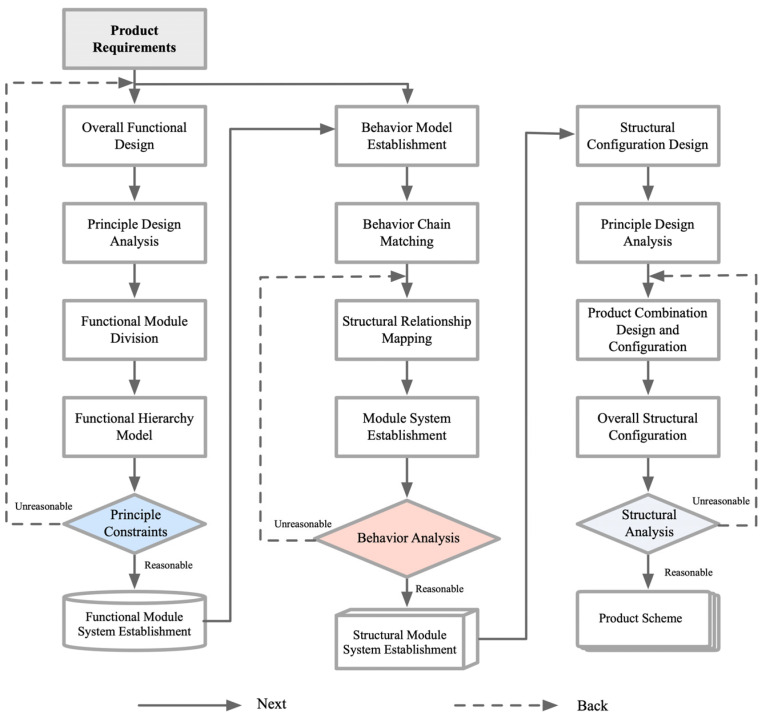
Product model conversion chart.

Viewing design modeling as a generalized configuration problem, this study proposes a design framework based on an ontology process knowledge representation approach [39]. The model consists of five basic elements, i.e., requirements, functions, principles, behaviors, and structures. The design goal is not only to capture the key information in the design process, but also to prompt the designer to think deeply and optimize the complex relationship between each element in the design process, so as to generate the product structure system. This is shown in Figure 4.

#### 3.1.2. RFPBS Design Process Modeling

**Figure 5:**
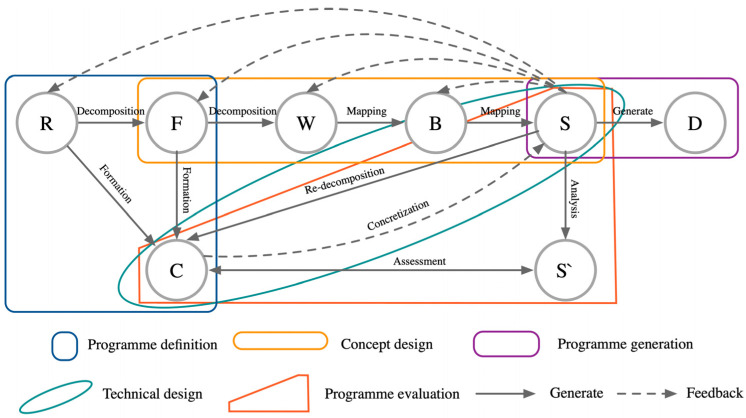
RFPBS mapping generation model.

The RFPBS mapping generation model was constructed through the above analysis, and the process was constructed through a series of interrelated stages and feedback mechanisms, as shown in Figure 5.

In this model, design begins with the Requirements Analysis (R) phase, which involves identifying and defining the project’s basic requirements. These requirements are then translated into specific Functional Objectives (F), a key step in transforming the design into actionable objectives. Functional goals are further refined in the Principle Space (P) phase, when the actual physical implementation is considered, and Behavioral Analysis (B) phase predicts and evaluates the performance of the prototype. The Design Structure (S) phase establishes the product-specific specifications and implementation requirements, which ultimately lead to the actual outputs of the design (D). Key dynamics in this model include constraints (C), which serve as guiding conditions throughout the design process, and system structure (S′), which defines the framework for design execution. Feedback mechanisms are clearly represented through direct and indirect paths between stages, symbolizing the two-way flow of information and the iterative nature of decision-making in the design process. Overall, the model highlights the importance of continuous feedback and adjustment between design phases to ensure that the final design outcome meets user needs and fits within the set constraints.

**Figure 6:**
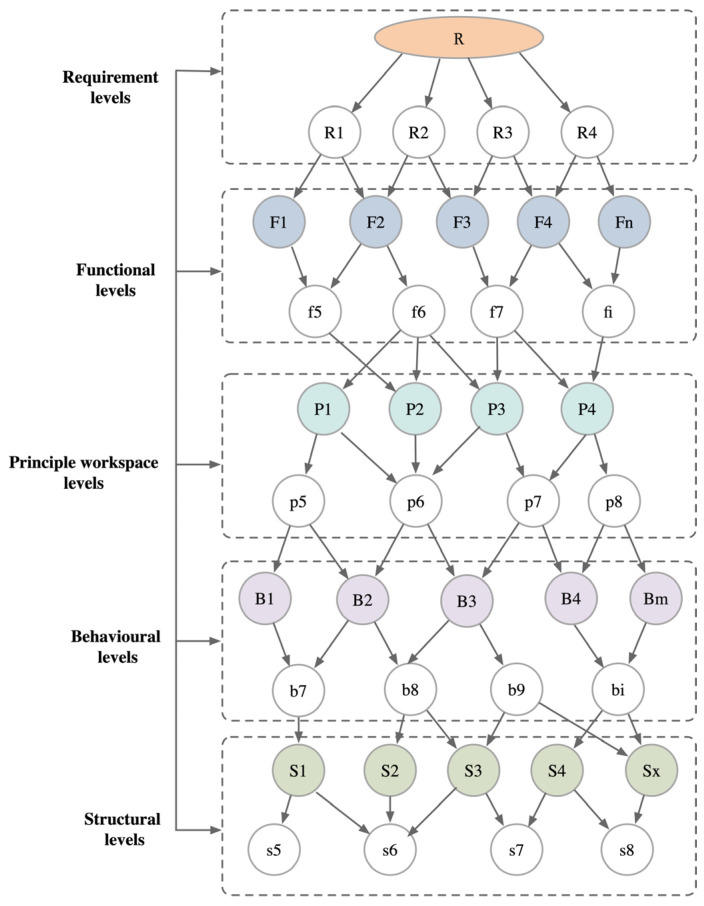
RFPBS mapping model.

In the discipline of engineering design, product design is viewed as an inherently complex and iterative process characterized by the precision of requirements and specifications unfolding incrementally as design goals are progressively achieved. In order to facilitate accurate decision-making by designers, the development of computer-aided tools based on efficient knowledge representation is essential. The RFPBS model presents an iterative design process across multiple levels of requirements, functions, principles, behaviors, and structures. The principal workspace layer is introduced in the model as a mechanism to introduce design constraints. Following this, a complete design solution is developed by mapping the principal workspace layer to the behavioral layer, which is then translated to the structural layer. By establishing a closed-loop mapping strategy and an explicit hierarchical structure, this study provides an adaptive process framework for knee exoskeleton design, as shown in Figure 6.

### 3.2. Multidimensional Analysis of the Knee Exoskeleton

#### 3.2.1. User Requirements Analysis

There is a causal relationship between needs and functions that needs to be explicitly described to facilitate knowledge reuse. The target population selected for this user study was stroke patients suffering from knee motion dysfunction, the patients’ family members, and the healthcare staff in the relevant rehabilitation department. Since it is more difficult to reach the target population in ordinary life scenarios, field research was conducted in the rehabilitation department of the hospital and the patients’ homes, in addition to the literature study conducted during the pre-study process. Using the KJ (affinity diagram) method to summarize and categorize the various types of information obtained from the observation method and the interview method, a large number of the expressed texts are converted into a clearer structural framework, as shown in the table. The structural framework divides user needs into two dimensions, namely, the basic needs dimension and the auxiliary needs dimension, and a total of eight user needs are identified; for ease of representation, they are labeled as Ri (where i = 1, 2, ⋯, 8). Among them, the basic needs dimension contains four needs: safety and stability, neurological rehabilitation, human–computer interaction, and comfort; the auxiliary needs dimension contains four needs: easy to wear, assisted rehabilitation, stylized rehabilitation, and personalized needs, as shown in Table 2.

#### 3.2.2. Biological Principles Analysis

**Figure 7:**
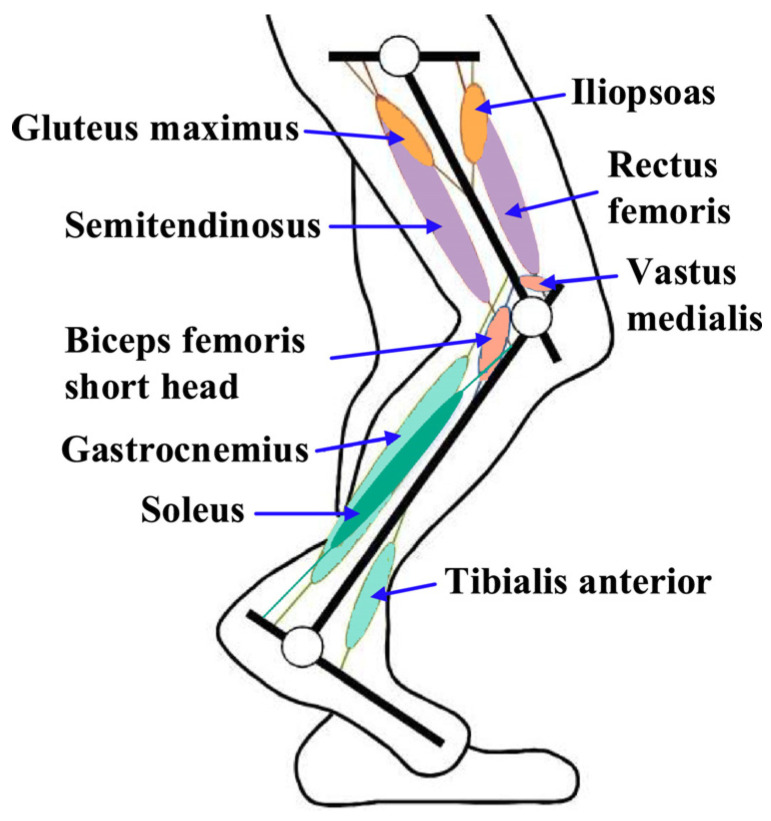
Human leg freedom and muscle distribution.

As a constraint in the process of complex product design, a principle is a bridge linking function and behavior, so it is necessary to analyze the principle of the human knee joint; doing so is conducive to the science and effectiveness of product module identification. The human knee joint is a structurally complex and functionally critical joint, composed of the femur, tibia, patella, and anterior and posterior cruciate ligaments, which can be modeled as a crossed four-bar linkage [40,41]. Its stability relies on the quadriceps muscle group, the medial and lateral collateral ligaments, and the anterior and posterior cruciate ligaments to maintain it. In addition, the shock-absorbing effect of the meniscus is critical for knee protection. In a normal gait, knee extension is carried out by the quadriceps muscles (rectus femoris, intermediate femoris, lateral femoris, and medial femoris), whereas knee flexion relies on the synergistic action of the semitendinosus, biceps femoris, semimembranosus, thin femoris, sutures, hamstrings, gastrocnemius, and metatarsal muscles. Figure 1 illustrates the activity of the lower limb muscles in the sagittal plane, which plays a crucial role in the coordination of gait movements, involving major muscles including the iliopsoas, gluteus maximus, rectus femoris, semitendinosus, vastus medialis, short head of the biceps femoris, gastrocnemius, soleus, and tibialis anterior muscles, as shown in Figure 7.

#### 3.2.3. Characteristics Analysis

**Figure 8:**
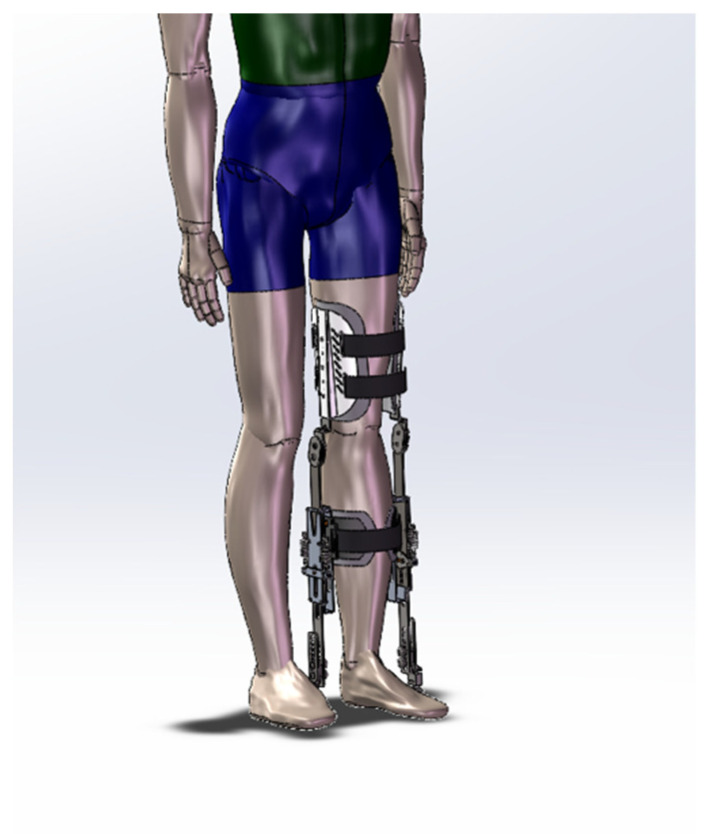
Exoskeleton diagram of the knee joint.

The design features of the knee exoskeleton were derived from the above analysis. The design features of the knee exoskeleton include low mass, connectivity that ensures comfort for the wearer, safety, and the dynamic assistive force and motion control features of the smart controller. This device connects to the wearer’s lower extremities through braces and straps, creating an exoskeleton system that works in concert with the human body. In this system, the knee exoskeleton moves in synchronization with the wearer’s knee, ensuring interaction between the two. The use of soft cushions further enhances the comfort and physical interaction between the wearer and the exoskeleton. The resulting knee exoskeleton structure is shown in Figure 8.

#### 3.2.4. Structural Model Construction

**Figure 9:**
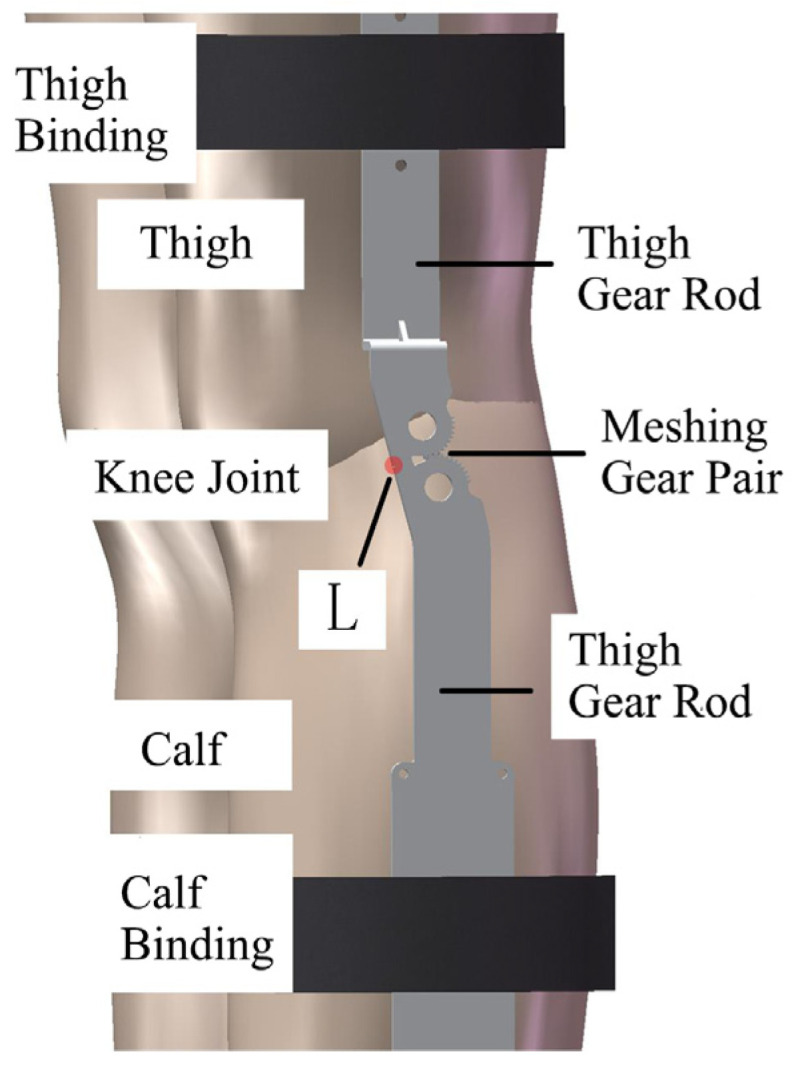
Schematic diagram of the knee exoskeleton mechanism.

The exoskeleton mechanism mainly includes the thigh gear lever, calf gear lever, and large and small leg bindings, of which one end of the thigh gear lever and calf gear lever is an incomplete gear structure which simulates the knee joint’s variable transient motion characteristics through the pair of meshing gears. The point L in Figure 9 is the hyperextension limit structure, which is realized by designing a right-angle transition at one end of the gears of the thigh gear lever and the calf gear lever that prevents the hyperextension of the knee joint when the knee joint is in extension and plays a role in protecting the knee joint for patients with hyperextension of the knee joint. The thigh gear lever and calf gear lever are parallel but not overlapping, so that they can effectively fit the natural curve of the limb.

#### 3.2.5. RFPBS Mapping Mechanism Established

**Figure 10:**
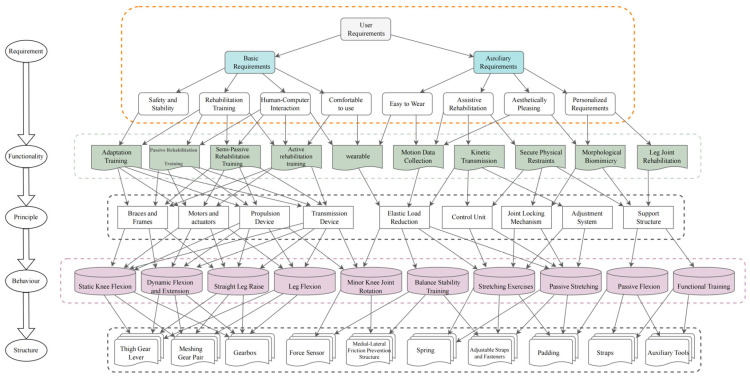
Knee rehabilitation exoskeleton RFPBS mapping framework.

Through the refined user requirement analysis, the precise mapping from requirement to function is derived to promote the development of the knee exoskeleton’s design. Firstly, the various modular units of the knee exoskeleton system are systematically identified, and then, based on their coupling strengths, the systematic reconstruction of functions, the establishment of principle models, the classification of behaviors, and the innovative construction of structural models are implemented in order to come up with the most optimized design solutions. At the abstraction level of functionality, key categories such as elastic load reduction, assisted rehabilitation, wearability and motion monitoring functions were identified through requirement and function mapping. Behavioral units were divided into basic behavioral units and integrated behavioral sequences, where the integrated behavioral sequences included a series of movement patterns, such as assisted force, bending, and extension, and the principle of behavioral compatibility was applied in order to achieve isomorphic mapping between movement features. In the structural dimension, a hierarchical model of the structure was established by analyzing the interrelationships and attributes of the components, and the structural component layer was divided into geared knee joints, functional electrical stimulation components, anti-friction design of the inner and outer knee joints, DC motors, etc., whereas the structural relationship level included myoelectric activity extraction sensors, force feedback sensors, and so on. Finally, based on the Requirement–Function–Principle–Behavior–Structure (RFPBS) design iterative deconstruction model, the multi-level mapping resolution of the knee rehabilitation exoskeleton is completed, as shown in Figure 10.

**Figure 11:**
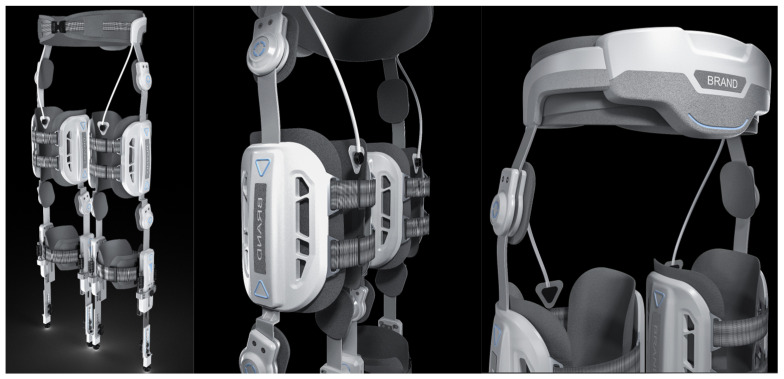
Knee exoskeleton design rendering.

The RFPBS mapping solution for the knee rehabilitation exoskeleton resulted in the final design shown in Figure 11. Traditional orthoses correct the knee joint through lateral force, which may lead to excessive wear and tear of the contralateral knee joint in long-term use. The knee decompression principle adopted in this design directly reduces the pressure on both sides of the knee joint, which has a better load-reduction effect, and the brace is designed with an adjustable structure to adapt to different patient sizes and improve its personalized fit. In addition, the design of the brace takes into account the comfort and safety of the calf, allowing the brace to slide freely relative to the calf and reducing direct forces. This knee exoskeleton system with integrated functional electrical stimulation provides efficient knee rehabilitation for paralyzed patients through the synergy of DC servomotors and electrical stimulators. The design demonstrates compactness, a light weight, and safe non-impact operation on the knee joint, marking an innovative advancement in the design of rehabilitation aids.

## 4. Results

### 4.1. Mechanical Effects of Knee Exoskeletons on the Human Lower Extremity

**Figure 12:**
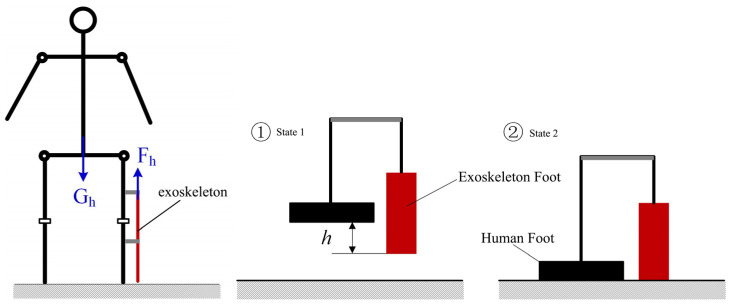
Schematic diagram of the principle of knee joint exoskeleton protecting the knee joint.

An important measure to protect the knee joint from injury is to reduce the pressure on the knee joint, and the mechanics of using a knee exoskeleton to protect the knee joint are shown in Figure 12. Worn on the sides of the limb, the exoskeleton does not touch the ground; as such, between the exoskeleton’s foot and the human foot there is a certain height difference, *h.* When the human foot descends from the air to gradually touch the ground (from step ① to step ② of the process), the spring deformation of the exoskeleton, due to the presence of two elastic coefficients of the same spring on the knee exoskeleton, produces an anti-gravity elastic force, *Fh*, which can be expressed as

(1) $$Fh=\left\{\begin{array}{c} 2K(l-l_{0})-m_{e}g,\;\;l\geq \frac{m_{e}g}{2K}+l_{0}\;\;\;\;\\ -m_{e}g,\;\;l<\frac{m_{e}g}{2K}+l_{0} \end{array}\right.$$

where *K* is the elasticity coefficient of the spring, *m_e_* is the mass of the exoskeleton, *g* is the acceleration of gravity, *l* is the length of the spring, and *l*_0_ is the original length of the spring. Assuming that the body weight of the human body is *G_h_*, when the human body stands, the two sides of the legs each share half of the body’s gravity. When wearing the knee exoskeleton on the lower limb on one side of the body, due to the existence of this anti-gravity elasticity, the pressure on that knee joint will be smaller compared to that on the knee joint on the side not wearing an exoskeleton. When wearing the exoskeleton on the lower limb on one side of the body, the knee joint pressure *f_p_* can be expressed as

(2) $$f_{p}=\frac{G_{h}}{2}-F_{h}\;\;\;\;$$

### 4.2. Simulation Analysis

The movement process from step ① to step ② can be regarded as the process of compression of the exoskeleton, and the spring located on the exoskeleton is stretched in the process, and the mechanical influence of the exoskeleton on the human body in the process can be simulated and analyzed. The exoskeleton weighs 1.85 kg, the elasticity coefficient of the spring is 2 N/mm, the original length of the spring is set to 50 mm, and the displacement of the spring is 40 mm. The lower limbs are in the supported state when the human leg takes a step and transitions to the foot touching the ground. The initial height difference, *h*, of the motion process is set to be 40 mm, the simulation time *t* is 2 s, and the drive displacement function is expressed as

(3) $$P=h\sin\left(\frac{\pi }{2}t\right)\ldots \ldots$$

**Figure 13:**
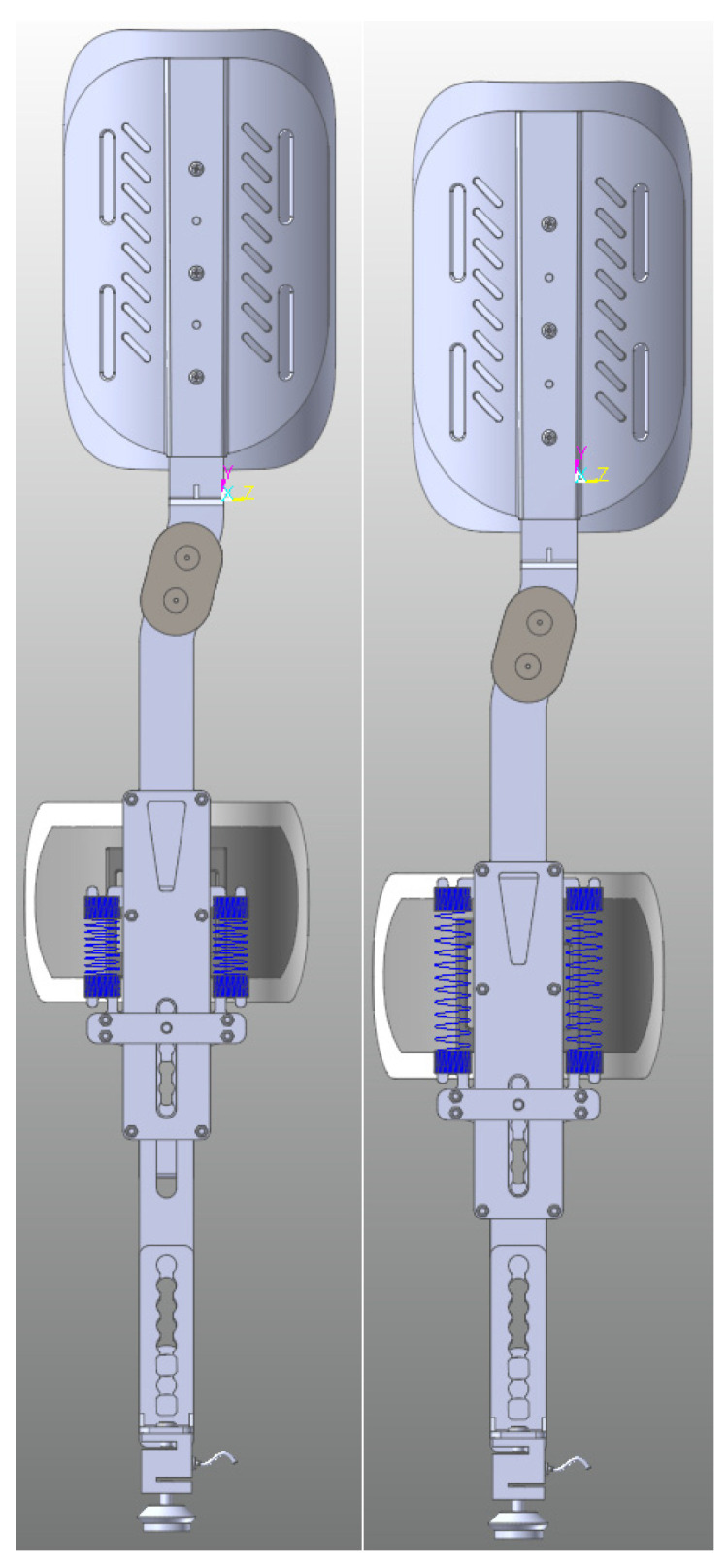
Initial state and maximum extension state of spring.

**Figure 14:**
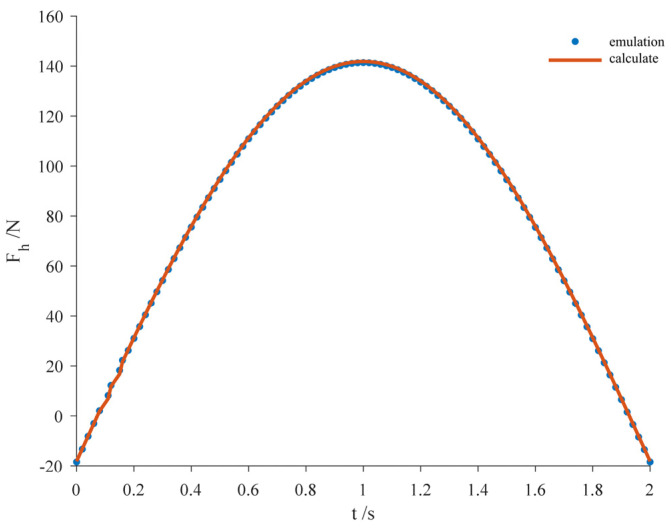
Simulation and theoretical calculation results of the elastic force in the anti-gravity direction generated by the exoskeleton.

After setting the simulation parameters in the Recurdyn v9r5 dynamics simulation software, the simulation is carried out, and Figure 13 and Figure 14 show the state demonstration during the simulation process and the change curve of the elastic force, *Fh*, during the whole movement process, respectively. When the spring is in the state of maximum elongation (*l* = 90 mm), the simulation time is 1 s; at this time, the exoskeleton produces a maximum elastic force of 141.4 N in the direction of anti-gravity on the human body, and the maximum elastic force, calculated according to Equation (1), is 141.8 N, which is approximately the same as the theoretical results, and the overall observation is that the curve of the theoretically calculated elastic force and the curve of elastic force obtained by the software simulation in Figure 14 coincide with each other, indicating that the equations reflect the mechanical effect of the exoskeleton on the human lower limb, thus verifying the correctness of the results of the theoretical analysis of the knee exoskeleton and the effectiveness of the exoskeleton in reducing the pressure on the human knee joint.

## 5. Discussion

**Figure 15:**
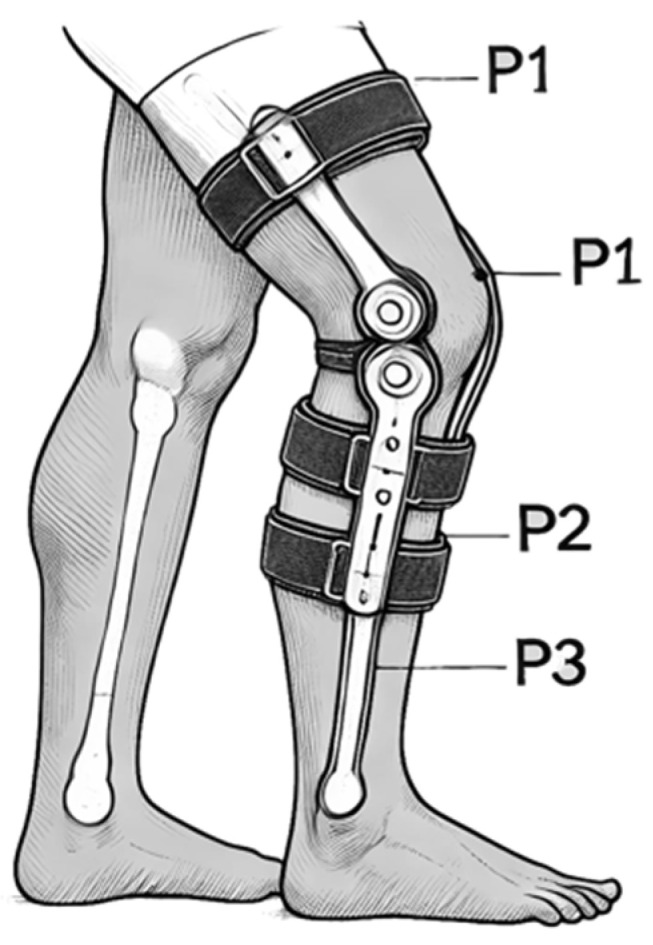
Traditional three-point mechanical knee orthosis. P1: This point is located on the thigh, above the knee. It mainly provides downward force, helping to stabilize the knee and resist upward forces. P2: This point is located on the lower leg, below the knee. It provides upward support, working in opposition to P1 to stabilize the knee. P3: Located on the opposite side of the knee (usually the outer or inner side of the leg), it offers additional stability, assisting in the control of lateral movement of the knee.

The working principle of traditional knee orthoses is based on a three-point pressure system, designed to stabilize or correct deformities in the knee joint. This design employs two force points on one side to apply pressure, while a third point on the opposite side helps create a mechanical balance, aiding in the correction of the position and movement of the knee joint. The three-point mechanical knee orthosis effectively disperses stress around the knee joint, thus protecting the joint, alleviating pain, preventing further damage, and promoting proper knee movement. This type of orthosis is commonly used for treating fractures of the knee joint, post-operative recovery, arthritis, and similar conditions, as shown in Figure 15.

In existing products like the Breg Roadrunner Knee Brace, the lateral force transmission in knee orthoses like this typically occurs from one side of the brace to the other. This transfer of force helps to correct lateral deviations or instabilities in the knee joint. Specifically, lateral force can be transferred from a support point on the inside of the brace to a braking point on the outside, or vice versa, to stabilize the knee joint and reduce lateral movement.

Long-term use of existing knee orthoses can lead to some issues. This is primarily because unloading braces transfer pressure from the diseased side (usually the side with more severe damage to the medial or lateral joint surfaces) to a healthier joint surface, thereby alleviating pain and wear on the affected side. However, this transfer of pressure may cause the following problems:

(1) Overload on the non-diseased side: As joint pressure decreases on one side it relatively increases on the other side. This increased load can accelerate wear on the non-diseased side of the joint, which could lead to the development of arthritis or other joint issues over time.
(2) Muscle imbalance and joint alignment issues: Long-term reliance on an orthosis can lead to changes in the strength and range of motion of leg muscles. Muscle strength imbalances may further affect joint stability and alignment, increasing the risk of injury.
(3) Biomechanical changes: Gait alterations caused by reliance on an orthosis can lead to changes in the force lines through the entire lower limb and spine. These changes can have a cascading effect on the entire musculoskeletal system, impacting the health of other joints and muscle tissues.

Differences between the orthosis proposed in this study and existing orthoses include:

(1) Traditional three-point mechanical knee orthoses, which pry open the affected side of the knee, can exacerbate wear on the other side over long-term use and are not suitable for prolonged use. The orthosis proposed here utilizes a more direct and effective knee joint decompression principle that directly reduces pressure on both sides of the knee joint, offering more apparent load-reduction effects and causing no knee joint damage over long-term wear;
(2) The brace features multiple adjustable structures, allowing for customization according to different patient needs;
(3) Compared to traditional knee orthoses, this brace allows for free sliding relative to the lower leg. The spring force is not applied to the lower leg but instead transfers body weight from the thigh to the ground, providing more direct knee joint decompression and increased comfort and safety at the lower leg;
(4) It uses a spring to alleviate joint pressure, with a compact structure and lightweight design, ensuring no impactful forces on the knee joint, making it safer.

## 6. Conclusions

The construction of the RFPBS process knowledge representation model provides an optimal matching solution between function and structure for the design of a knee exoskeleton for gait rehabilitation. The model integrates multi-dimensional considerations such as user requirements, functional design, principal constraints, behavioral simulation, and structural implementation, which in turn supports the iterative design process of the knee exoskeleton. The simulation results show that the proposed model can effectively simulate the biomechanical behavior of the knee joint, further optimize the structural design of the knee exoskeleton, and play a key role in reducing the burden on the knee joint and assisting rehabilitation training. Compared with existing studies, this study examines the problem of knowledge representation in the design process from a new perspective, highlights the importance of integrating multidimensional models in the design of complex systems, and proposes new guiding principles for combining theory with practical applications. Future work will focus on further validation and optimization of the model, especially deepening the effectiveness of the model in practical rehabilitation training, and continuous iterative improvement through user feedback.

## Figures and Tables

**Table 1:** Comparison of various design modeling studies.

Model Type	Description of Subject	Knowledge Representation	Background Knowledge of Design	Multi-Dimensional Knowledge Integration	Computer-Aided Applicability
FBS [34]	Function	Design objects and their relations	No	N/A (Not applicable)	Moderate
	Behavior				
	Structure				
Situated FBS [35]	Situational Ffunction	Design knowledge transformation	No	N/A	N/A
	Behavior				
	Structure				
RFBS [36]	Requirement	Model-driven and integrated	Yes	N/A	Moderate
	Function				
	Behavior				
	Structure				
FCBS [37]	Function	Understanding, representing and reusing present design knowledge	No	N/A	Moderate
	Cell				
	Behavior				
	Structure				
RFPBS	Requirement	Design knowledge multi-level hybrid mapping solution	Yes	Yes	High
	Function				
	Principle				
	Behavior				
	Structure				

**Table 2:** User requirements dimension.

User Requirements Dimension	Serial No.	User Requirement
Basic needs	R_1_	Security and stability
	R_2_	Rehabilitation
	R_3_	Precise human–machine interaction
	R_4_	Comfortable to use
Subsidiary needs	R_5_	Easy to wear
	R_6_	Supported rehabilitation
	R_7_	Aesthetic design
	R_8_	Individualized needs

## Data Availability

No new data were created or analyzed in this study. Data sharing is not applicable to this article.

## Abbreviations

FBS	Function–Behavior–Structure
Situated FBS	Situational Function–Behavior–Structure
RFBS	Requirement–Function–Behavior–Structure
FCBS	Function–Cell–Behavior–Structure
RFPBS	Requirement–Function–Principle–Behavior–Structure
D	Design
C	Constraints
S′	System Structure
KJ	Kawakita Jiro
